# The Transition to Winter

**Published:** 1901-10-12

**Authors:** 


### The Hospital

A Journal of

The Medical Sciences and Hospital Administration.

Vol. XXXI.—No. 785.] Edited by Sir Henry Burdett, K.C.B., and Solomon C. Smith, M.D., M.R.C.P. [October 12, 1901.

### The Transition to Winter.

The advent of "chill October," which has this year been accompanied by alternate days of fine and of boisterous weather, is a period which, in all seasons and in all weathers, calls for some consideration at the hands of those prudent people who desire to carry onward into winter the health and vigour which have been reinforced by their autumnal holiday. In old times, before the multiplication of railways had facilitated the interchange of commodities, and when every district was primarily dependent upon the products of its own locality for the sustenance of its inhabitants, it was a matter of obvious necessity to live chiefly upon salted provisions during the winter months, when fresh meat was only procurable in very limited quantities, and when fresh vegetables could not be procured at all; and so the wisdom of our ancestors taught them that the half-yearly change of diet should be prepared for, in anticipation alike of winter and of summer, by some form of simple domestic medication, or, not unfrequently, by blood-letting, which was then constantly undergone by perfectly healthy people, as a purely precautionary measure, at both "spring and fall." Increased facilities for the winter feeding of cattle, the growth of winter vegetables, and other obvious causes gradually removed all occasion for the changes of diet which had once been compulsory; the bleeding and physicking fell by degrees into desuetude. Uniformity of diet, however, does not convert the approach of winter into the approach of spring, and it is by no means uncommon for the former season to be heralded by what people are accustomed to call "taking cold" in such a fashion that more or less *malaise* and incapacity are carried forward from October or November for many months to come. It is surely the part of a wise man to say to himself at this season of the year, "My life is about to be carried on under somewhat modified conditions. In what way may I best adapt myself to them?

The period of "spring and fall" medication was succeeded by another in which the aforesaid "taking cold" was regarded as the evil to be chiefly dreaded and guarded against, and when "draughts" were believed to be the agencies by which the deleterious capture might most readily be brought about. The consequence was an age of stuffiness, founded upon beliefs which still dominate the muddled brains of people who would exclude ventilation from railway carriages and who afterwards wonder at the ill-success which has attended upon their "precautions." In the present day, when a knowledge of the influence of pure air and sunlight upon the tubercle bacillus is beginning to be diffused abroad, people in general are perhaps a little apt to go into the opposite extreme, and to carry their desire for the former of these agents to an extent which compels them to neglect the perhaps equally important element of temperature. It is probable that the majority of what are called minor ailments are due to the invasion of the body by some bacillus which, under a given set of conditions, is able to resist the agencies by which, in other conditions, it would be overcome; and, if so, it can hardly be doubted that reduction of external temperature, long fasting, a vitiated atmosphere, and over fatigue, are prominent among the conditions which tend to turn the scale against the invaded organism and in favour of the invader. The fall of the outdoor thermometer is so far an indication for an early increase in the thickness of the underclothing, and for care that it is made either of silk or wool or of an admixture of the two, in accordance with the sound principle laid down long ago by the late Sir Francis Bond Head, to the effect that the clothing of animal bodies, in these latitudes, should be made from materials derived from the animal kingdom. In this respect, as in most others, and as a matter at once of prudence and of policy, the preference should be given to textures of English manufacture; and care should be taken to avoid what, in the esoteric jargon of the more dishonest portions of "trade," are called "plated goods," that is to say, fabrics woven from a cotton yarn, thinly covered and concealed by a layer of silk or wool. An unravelled fibre under the microscope will at once display the cheat. Perhaps the most critical stage of the transition period is that which comes before fires are universal in the dwelling-rooms, and when a man who returns home tired for his dinner may have to wait half an hour or so for it in a chilled apartment. A change of foot-gear should at least be regarded as essential to security in such circumstances; and it is probable that half a glass of sound wine, taken on arrival, would frequently act as a preservative against ill-consequences. Details, however, must of necessity be governed by circumstances; and the real object of our sermon is to enforce the truth that different external conditions must be met in the ways respectively suited to them, and that the dress and habits of summer may become sources of danger when a more inclement season is at hand.

